# Macrophage orchestration of epithelial and stromal cell homeostasis in the intestine

**DOI:** 10.1002/JLB.3RU0322-176R

**Published:** 2022-05-20

**Authors:** Qian Cao, Randall Tyler Mertens, Kisha Nandini Sivanathan, Xuechun Cai, Peng Xiao

**Affiliations:** ^1^ Department of Gastroenterology, Sir Run Run Shaw Hospital Zhejiang University School of Medicine Hangzhou China; ^2^ Inflammatory Bowel Disease Center, Sir Run Run Shaw Hospital Zhejiang University School of Medicine Hangzhou China; ^3^ Department of Immunology Harvard Medical School Boston Massachusetts USA; ^4^ Evergrande Center for Immunologic Diseases Harvard Medical School and Brigham and Women's Hospital Boston Massachusetts USA; ^5^ The Key Laboratory for Immunity and Inflammatory Diseases of Zhejiang Province Hangzhou China; ^6^ Institute of Immunology Zhejiang University School of Medicine Hangzhou China

**Keywords:** intestinal epithelial cells, intestinal inflammation, macrophages, mucosal immunity, stromal cells

## Abstract

The intestinal tract is a complex ecosystem where numerous cell types of epithelial, immune, neuronal, and endothelial origin coexist in an intertwined, highly organized manner. The functional equilibrium of the intestine relies heavily on the proper crosstalk and cooperation among each cell population. Furthermore, macrophages are versatile, innate immune cells that participate widely in the modulation of inflammation and tissue remodeling. Emerging evidence suggest that macrophages are central in orchestrating tissue homeostasis. Herein, we describe how macrophages interact with epithelial cells, neurons, and other types of mesenchymal cells under the context of intestinal inflammation, followed by the therapeutic implications of cellular crosstalk pertaining to the treatment of inflammatory bowel disease.

## Graphical Abstract

This review focuses on how macrophages regulate intestine inflammation through cross‐talking with epithelial cells and mesenchymal cells in the gut.

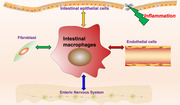

**Abbreviations**
AMP: antimicrobial peptides
CAIP: cholinergic anti‐inflammatory pathway
DAMP: danger‐associated molecular patterns
DC: dendritic cell
DSS: dextran sulphate sodium
ENS: enteric nervous system
IBD: inflammatory bowel disease
IEC: intestinal epithelial cells
ILC: innate lymphoid cell
IMφ: intestinal macrophage
MDSC: myeloid‐derived suppressor cells
MSC: mesenchymal stem cells
NLRs: NOD‐like receptors
ROS: reactive oxygen species
SP: Substance P
TED: transepithelial dendrites
UCMSC: umbilical cord mesenchymal stem cells
VIP: vasoactive intestinal peptide

## 1 INTRODUCTION

The mammalian intestine is a site where numerous external and internal signals constantly converge. Besides functioning as a digestive and absorptive organ, the intestinal tract can be seen as the largest peripheral immune organ, which harbors over 70% of the body's total immune cells.^1^ Macrophages belong to the mononuclear phagocyte system, densely populated throughout the intestinal lamina propria and found in close proximity to intestinal epithelial cells (IECs).^2^ These versatile immune cells are also widely distributed throughout the submucosa, muscularis externa, and serosa layers, where they receive signals from the enteric neurons and various mesenchymal cells. As such, macrophages play a pivotal role in generating feedback signals to orchestrate the functions of these neighboring cells. Dysfunction of intestinal macrophages (IMφs) is typically “infectious,” resulting in transmission of the wrong information to other cell types, consequently triggering a vicious cycle that ultimately destroys the intestinal equilibrium. Deeper insight into the mechanisms underlying macrophage‐mediated intercellular crosstalk is pivotal to the development of successful inflammatory bowel disease (IBD) therapeutic strategies. In this review, we summarize current knowledge about the reciprocal regulation between IMφs, IECs, and other varying stromal cell subtypes within the intestine and conclude by discussing its relevance to clinical therapeutic IBD intervention.

## 2 ORIGIN AND PHENOTYPE OF IMφS

The origins of tissue‐resident macrophages mainly include the yolk sac, fetal liver, and bone marrow^3^; however, the relative contribution of these sources varies greatly among different organs. In the steady state, brain macrophages (microglia) are almost exclusively of yolk sac origin after birth. Macrophages in other organs, such as the lung, liver, or epidermis are mostly derived from fetal liver monocytes.^3^, ^4^, ^5^, ^6^ In contrast, fate‐mapping analysis showed that the origin of IMφs were quite different, as they are constantly replenished by CCR2^+^ peripheral monocytes in the adult mouse.^7^ Monocyte infiltration into the steady‐state intestine is thought to be mediated through gut microbiota‐dependent “physiological inflammation.”^8^ Previous research has validated this dependency as the number of IMφs were greatly reduced in CCR2^–/–^ mice 1 week after birth, yet the number of liver macrophages was not affected.^7^ In support of this finding, CCR2‐DTR mice administered with diphtheria toxin exhibited a near complete loss of IMφs.^9^ Challenging previous findings, a subset of self‐renewing IMφs with a Tim‐4^+^CD4^+^ phenotype was reported.^10^ This specific subset of long‐lived IMφs is mainly localized in the muscularis and submucosa layers, and their accumulation in the intestine is independent of CCR2.^10^, ^11^

In general, IMφs express classical macrophage markers, including F4/80 and CD68, which are commonly used with the pan‐myeloid marker CD11b to define IMφs in many studies. On the other hand, unlike other tissue‐resident macrophages, a large proportion of IMφs express high levels of MHC‐II and CD11c, which are considered to be markers of dendritic cells (DCs). To date, various phenotyping strategies for IMφs have been proposed. A combination of MHC‐II^+^CD11c^+^CD64^+^ was suggested for identifying IMφs,^12^ based on the findings that CD64^+^ cells required M‐CSF for their development. Meanwhile, the development of CD64^–^ cells was dependent on Flt3L, a known DC growth factor.^13^, ^14^ Moreover, among the MHC‐II^+^CD11c^+^ population, CD64^+^ cells displayed typical morphologic features of macrophages and could not migrate to the mesenteric lymph nodes, indicating their macrophagic character.^12^, ^13^, ^14^ High consideration and caution should be taken when translating results/phenotypes from mouse models to human IMφs. For example, F4/80 and Ly6C, two classically used markers to identify mouse monocyte/macrophage linage, have no counterpart in humans. Furthermore, mature human IMφs have been reported as negative for CD11b, CD11c, and CD64. It is worth mentioning that these markers were highly expressed on peripheral monocytes from the same individual, illustrating the complexity involved when translating science from mice to human populations.^15^ Moreover, CX3CR1 expression was also identified to be low in human IMφs. A recent report identified four different macrophage subsets within the human small intestine, exhibiting distinct surface markers, turnover time, tissue localization, and gene expression profiles.^16^ Despite these phenotypic discrepancies, mouse and human IMφs in fact do share functional similarities. Both possess high phagocytic capacity and are refractory to the stimulation of pathogen‐associated molecular patterns (PAMPs).^16^

Phenotyping of IMφs is complicated further in the inflamed intestine, where massive amounts of blood monocytes are continuously recruited. Upon entering the intestine, these inflammatory monocytes undergo a so‐called “monocyte waterfall” to fully differentiate into mature IMφs. During this process, monocytes gradually lose Ly6C expression and acquire/up‐regulate the expression of MHC‐II/CX3CR1, respectively. Functional changes also occur after this infiltration process. Ly6C^hi^MHC‐II^–^CX3CR1^int/low^ (immature IMφs) produce high levels of IL‐6, iNOS, and IL‐23, whereas Ly6C^–^MHC‐II^+^CX3CR1^hi (^mature IMφs) mainly produce IL‐10 and express CD163 and CD206.^17^ Throughout the remainder of this review, different monocyte/macrophage subsets were indiscriminately described as “IMφs,” unless otherwise noted. Monocytes are further considered to be progenitors for mature IMφs, though a significant proportion fail to differentiate even under highly inflammatory conditions (as discussed later in the review).

Although macrophages are traditionally divided into “classically activated macrophages (M1)” or “alternatively activated macrophages (M2)” mirroring the CD4^+^ T helper cell “Th1/Th2” classification, this simple dichotomy may be far from precisely covering the diversity of IMφs. The presented examples demonstrate the need for researchers to delve deeper into understanding the subsets of IMφs, which contain extremely heterogeneous subsets according to their origin, location, and received environmental signals. Therefore, each IMφ subset may uniquely participate in intracellular communication and play a critical role in regulating intestinal inflammation.

## 3 CROSSTALK BETWEEN IECS AND IMφS

**FIGURE 1:**
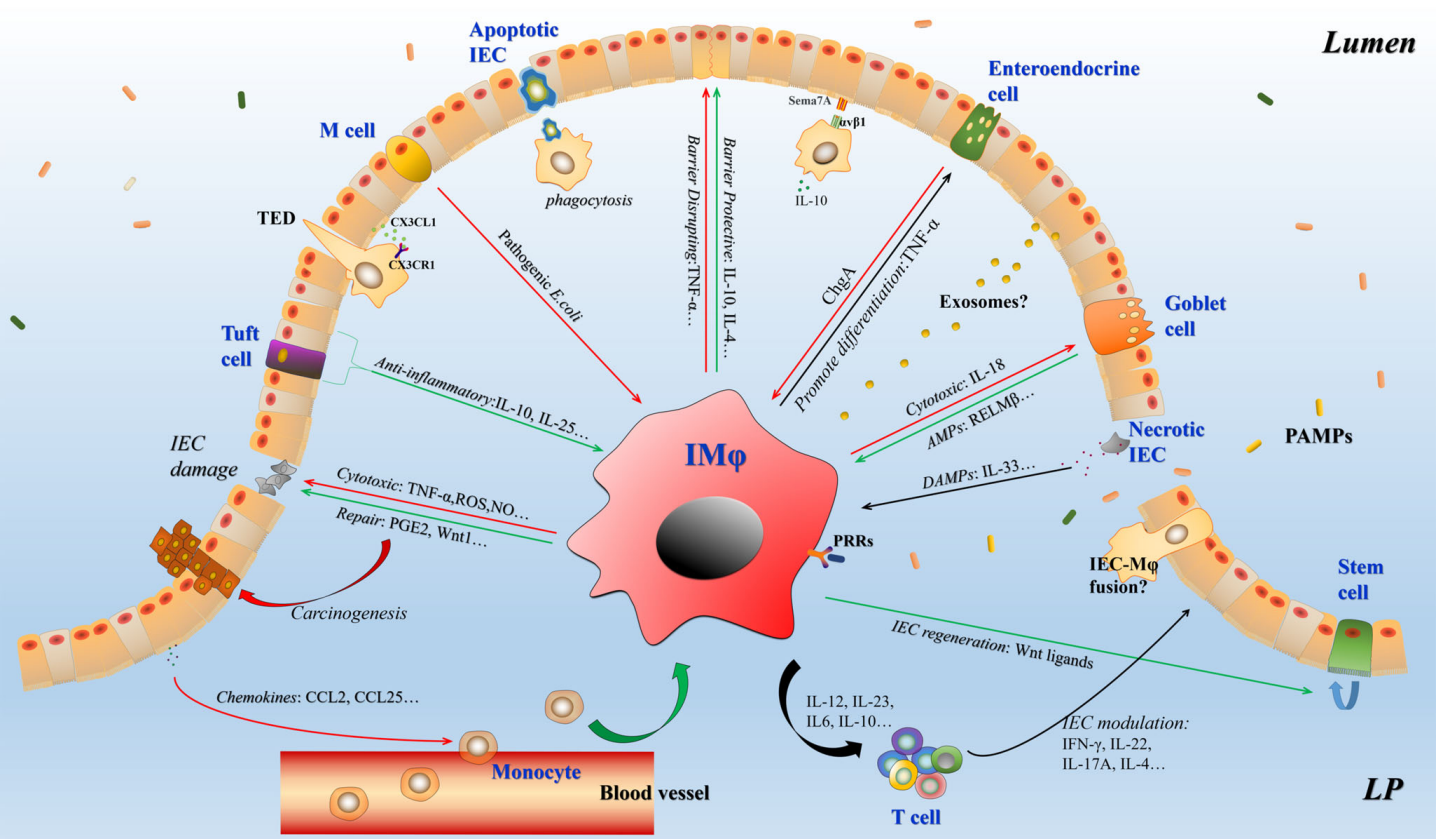
IEC–IMφ crosstalk. Green arrows represent anticolitic effects; red arrows represent procolitic effects; black arrows represent uncertain or multifaceted outcomes

IECs comprise the single cell layer lining the gut between the lumen and external environment.^18^ The fundamental functions of IECs are mainly attributed to nutrient absorption, barrier formation, and immune regulation. There are classified into several types of mature IECs including: enterocytes, paneth cells, goblet cells, enteroendocrine cells, M cells, and tuft cells.^19^, ^20^ Together, these cells form the epithelial barrier, which segregates the gut bacteria and lamina propria in order to prevent the activation of inappropriate immune responses. Due to the close proximity of IECs and IMφs, they frequently interplay in both the healthy and inflamed intestine (Figure 1).

### 3.1 Epithelium regulation of IMφ functions

First, IECs are important sources of monocyte‐attracting chemokines in intestinal inflammation. IEC‐derived TGF‐β and IL‐8 chemo‐attract peripheral monocytes into the intestinal mucosa.^21^ Also, CCL25 produced by IECs recruit CCR9^+^ monocytes to the inflamed intestine. By blocking physiologic CCL25/CCR9 interactions using CCL25‐conjugated Sepharose beads, intestinal inflammation was found to be alleviated in IBD patients by selectively deleting CCR9+ monocytes.^22^, ^23^ Similarly, in a clinical trial for ulcerative colitis, CCL25‐conjugated Sepharose beads have been found to decrease the number of circulating HLA‐DR^hi^ inflammatory monocytes with no obvious adverse side effect.^24^ Recently, IECs were found to serve as a major source of a novel CCR2 ligand: PC3‐secreted microprotein (PSMP), which mediates the infiltration of Ly6C^hi^ monocytes into colon, resulting in colitis development. The production of PSMP occurred prior to the up‐regulation of CCL2 in the inflamed colon, suggesting that IEC‐derived PSMP may be crucial for the early recruitment of inflammatory monocytes.^25^ Furthermore, IEC‐derived MMP9 facilitated the infiltration of CD11b^+^ inflammatory monocytes, which induced colonic mucosa damage.^26^

Upon entering the inflamed mucosa, IMφs receive signals from IEC‐derived cytokines.

IECs express a wide range of pattern recognition receptors (PRRs), such as TLRs and NOD‐like receptors (NLRs). Through these PRRs, IECs can then actively sense various kinds of bacterial stimuli and subsequently produce immunoregulatory cytokines.^27^, ^28^ For example, LPS (a TLR4 ligand)‐stimulated IECs serve as important sources of mucosal IL‐10 and TGF‐β, two critical immunosuppressive cytokines responsible for suppressing macrophagic production of inflammatory cytokines. This further implies a significant IEC contribution to the anti‐inflammatory programming in IMφs.^29^, ^30^ Aside TLRs, IEC‐expressed NLRs are also clinically relevant to IBD pathogenesis due to their key role in inflammasome activation. Polymorphisms in NOD2, an intracellular NLR that recognizes diaminopimelic acid‐containing muramyl tripeptide or muramyl dipeptide from bacterial peptidoglycans, is closely linked to genetic risk for Crohn's disease. Furthermore, IEC‐intrinsic NLR‐inflammasome signaling has profound impacts on the intestine immune system.^31^ An important event downstream of inflammasome activation is to release mature IL‐1β and IL‐18. Different from myeloid cells, IEC do not produce significant levels of IL‐1β upon inflammasome activation.^32^ In contrast, IECs were the primary source of IL‐18.^33^, ^34^ It is worth mentioning exactly how IL‐18 impact macrophage functions remains controversial. IL‐18 was also reported to promote TNF‐α secretion from macrophages.^35^ Consistently, IL‐18 neutralization reduced TNF‐α production in colitic mice.^36^ However, IL‐18 amplified the anti‐inflammatory phenotype of macrophages induced by IL‐10.^37^ Hence, the impacts of IEC NLR signaling on macrophage functions still need further elucidation. Other IEC‐derived cytokines with macrophage‐modulatory function include thymic stromal lymphopoietin, which promoted the polarization of M2 macrophages,^38^ thus inhibiting intestinal inflammation and promoting tissue repair. IEC‐secreted FNDC4, a fibronectin type III domain‐containing protein, also exerts an anti‐inflammatory function by suppressing the production of inflammatory chemokines in IMφs.^39^ Compared with IECs from healthy mucosa, IECs from patients with IBD expressed a markedly higher level of IL‐37,^40^ which was implicated in the protection against dextran sulphate sodium (DSS) colitis.^41^ The anticolitic effect of IL‐37 may be partially attributed to its ability to down‐regulate the production of inflammatory cytokines in macrophages.^42^

The inflammatory intestinal microenvironment poses profound stress on IEC survival. This inflammatory stress ultimately leads to the release of danger‐associated molecular patterns with immunomodulatory properties. A typical example is IL‐33, an IL‐1 cytokine family member that is predominantly expressed in nonhematopoietic cells.^43^ In a murine colitis models, IL‐33 was reported to ameliorate disease progression by increasing M2 macrophage polarization, or by promoting macrophage autophagy.^44^, ^45^ However, there was also a contradictory report illustrating IL‐33 administration aggravated DSS colitis by amplifying Th2 response and increasing the number of IMφs.^46^ More interestingly, IL‐33 was also reported to either promote or impair mucosal restitution and healing in two separate studies.^47^, ^48^ The seemingly contradictory results mentioned above may suggest that the protective role of IL‐33 requires a homeostatic balance within the gut. Insufficient or excessive production of IL‐33 will lead to the exaggerated inflammation. The in vivo function of IL‐33 is further complicated by the fact that it can be cleaved by various extracellular and intracellular enzymes to generate truncated forms with different bioactivity.^49^, ^50^, ^51^ It should be noted that although many IEC‐derived cytokines/soluble factors are also produced by other cell types. In some cases, IECs might be the predominant sources of these mediators due to their high cell number in the intestine.

Different IEC subsets have unique manners to regulate IMφ function. For example, serotonin produced by a subset of enteroendocrine cells (enterochromaffin cells) contributes to colitis development by increasing the infiltration and inflammatory activity of IMφs.^52^ On the other hand, through producing chromofungin—a short peptide derived from Chromogranin‐A proteolytic processing—enteroendocrine cells enhanced the alternative activation of IMφs, resulting in the amelioration of murine DSS colitis.^53^ Goblet cells, another IEC subset, are intestinal secretory cells whose main function is to synthesize and secrete mucins and antimicrobial peptides (AMPs). Goblet cell‐specific AMP RELMβ up‐regulated the expression of TNF‐α, IL‐12/23p40 and MHC‐II in macrophages, facilitating the establishment of a Th1‐dominant immune response. This unique phenomenon exacerbated intestinal inflammation induced by chronic *Trichuris* infection.^54^ In DSS colitis, macrophages from RELMβ^–/–^ mice exhibited lower levels of TNF‐α and IL‐15 production, resulting in mice more resistant to intestinal inflammation. Even though RELMβ is highly expressed in goblet cells, RELMβ deficiency did not obviously affect epithelial barrier function.^55^

In recent years, a specialized IEC subtype—tuft cells—have been shown to modulate intestinal immunity.^56^ Tuft cells are the predominant sources of IL‐25 in both the healthy and helminth‐infected intestine, by which they promote a Th2 response.^57^ It is possible that tuft cells can modulate intestinal inflammation via affecting IMφ functions, as IL‐25 has been reported to alleviate colitis by reducing the inflammatory capacity of macrophages^58^ and inducing the polarization of alternatively‐activated macrophages.^59^ Conversely, in a type‐2 colitis model induced by oxazolone, IL‐25 signaling was shown to be pathogenic by enhancing the production of IL‐13, a major epithelium‐toxic cytokine. It is essential that the exact role of IL‐25 in colitis needs further investigation. Microfold cells (M cells) are an additional form of specialized IECs whose main function is to sample luminal antigens and transport them to the subepithelial lymphoid follicles. This transport is done in order to initiate immune responses in GALT.^60^ It has been shown that M cells uptake then transfer enterohemorrhagic *Escherichia coli* to IMφs, resulting in increased bacterial survival and induction of apoptosis of IMφ, ultimately leading to the release of Shiga‐toxins into the bloodstream.^61^

Besides the soluble factor‐mediated crosstalk, the proximity between IECs and subepithelial macrophages also allows them to interact in a contact‐dependent manner. Semaphorin 7A, expressed on basolateral IECs, binds to αvβ1 integrin on IMφs, thereby triggering macrophage production of IL‐10, which was shown to ameliorate colitis.^62^ Concurrently, macrophages project transepithelial dendrites (TEDs) outside of the IEC barrier to sample lumen bacteria.^63^, ^64^ This process depends on macrophage CX3CR1 expression. This coincides with a recent report that CX3CR1^–/–^ mice failed to form TEDs.^64^ IECs are thus more than likely involved in regulating the formation of TEDs as they express the sole known CX3CR1 ligand—CX3CL1.^65^ Despite this knowledge, the physiologic significance of TEDs in intestinal inflammation is poorly understood. Furthermore, to complicate matters, the presence of TEDs seems to depend on the particular mouse strains.^64^

Not only can IECs heavily influence IMφ in the living microbiome, interestingly, dead IECs have the potential to also shape IMφ function. Homeostatic apoptotic IECs were phagocytized by CD103^+/–^CD11b^+^CD24^–^CD64^+^ IMφs and CD103^+^CD24^+^CD64^−^ DCs in the small intestine. The recognization of dead IECs markedly changed gene expression profiles of IMφs, with a general up‐regulation in anti‐inflammatory genes and down‐regulation in proinflammatory genes.^66^

Another intriguing manifestation of IMφ‐IECs crosstalk is cell fusion. Bone marrow‐derived cells were reported to be able to fuse with various mature IEC lineages and intestinal stem cells in the injured intestinal mucosa.^67^ Similarly, it is reported that bone marrow‐derived cells can fuse with proliferating IECs in the intestine of IL‐10^–/–^ mice. This fusion effect was inhibited by treating IL‐10^–/–^ mice with anti‐inflammatory agent 5‐ASA, suggesting that this particular cell fusion phenomenon was driven by intestinal inflammation.^68^ Although the aforementioned studies did not specify which subpopulation of bone marrow‐derived cells participated in the fusion with IECs, following work illustrated that IMφ–IEC fusion was observed during the development of colon tumors. Crypt IECs, which were fused with IMφs, acquired not only the macrophage surface marker F4/80, but also a set of specific genes related to macrophage functions.^69^ These findings raise several interesting questions: (1) What is the physiologic significance of IMφ–IEC fusion in intestinal inflammation? (2) How does this process affect disease progression? (3) Which factors mediate this cell fusion and the underlying molecular basis? This cell fusion process resembles the uptake of extracellular vesicles, in which the recipient cells acquire certain characteristics of the donor cells. Indeed, IECs generate an abundant number of exosomes to modulate the function of immune cells, such as DCs.^70^, ^71^ Although direct evidence is lacking, it is reasonable to hypothesize that exosomes also contribute to IEC‐mediated IMφ regulation of intestinal inflammation. This leads to yet another interesting cell–cell dynamic that has not been deeply explored: why do cells need exosomes to convey information? The production of exosomes is an energy‐consuming process and the close proximity between IECs and IMφs inherently makes exosome production seem as an unnecessary biologic function. A plausible explanation could be that IECs release certain exosomes to deliver a specific “molecule combination,” rather than a set of randomly packaged molecules. Therefore, each component in the exosome package would act synergistically to fulfill a certain regulatory purpose.

### 3.2 IMφs communicating with IECs—feedback mechanisms

Macrophages are well accepted for their phagocytic and tissue‐remodeling abilities. In the homeostatic intestine, IMφs actively phagocytize the effete IECs within the intestinal villi to maintain epithelial turnover.^72^ When the IEC barrier is mechanically injured, IMφs accumulate around the wound bed and ensure effective epithelial healing.^73^, ^74^ In the literature, blood‐derived macrophages from healthy donors, or patients with IBD, displayed a CD206^+^CCL18^+^CD14^low/−^phenotype upon IL‐4 treatment, thus acquiring the ability to accelerate epithelial wound healing by producing TGF‐β.^75^ IL‐4‐primed macrophages were also found to secrete miR‐590‐3p‐containing exosomes, which then facilitated epithelial repair by activating the LATS1/YAP/β‐catenin pathway.^76^ Furthermore, in the inflamed gut, macrophages, which produced IL‐36, stimulated the proliferation and AMP production in IECs, thus facilitating the recovery of the damaged IEC barrier.^77^ Mesenchymal macrophages are also likely crucial for the establishment of an epithelial‐regenerative niche in the damaged colonic mucosa. This effect was found to be mediated through Myd88‐depedent production of several proregenerative mediators by macrophages in response to gut microbiota.^78^ IL‐10, although being previously thought as an immunosuppressive cytokine,^79^ was recently reported to exert a direct protective role on intestinal epithelium. Macrophage‐derived IL‐10 accelerated the repair of the injured colonic mucosa through CREB‐dependent WISP‐1 secretion. Also, the absence of IL‐10 signaling in IECs further impaired their proliferation and wound‐healing capacity.^80^ Moreover, in mice colonized with *Enterococcus*—a colitogenic bacteria—IL‐10 was reported to alleviate endoplasmic reticulum stress (ERS) in IECs by inhibiting the recruitment of ATF‐6 to the promoter region of GRP78—an ERS marker.^81^ Another form of macrophagic communication was revealed by the ability of M2 polarized macrophages to produce several isoforms of Wnt ligands, thus accelerating the mucosal repair in colitic mice via STAT6‐dependent mechanism.^82^ In addition, hypoxia stimulated macrophages to release Wnt1, which inhibited the autophagy of IECs located within the damaged mucosa by β‐catenin and mTOR signaling pathway activation.^83^ Similarly, M2 macrophage‐derived Wnt1 was shown to activate the Wnt/β‐catenin signaling in crypt IECs, leading to inhibition of IEC differentiation. This may result in promoting IEC proliferation and wound healing while concomitantly increasing the risk of colorectal adenocarcinoma.^84^ Due to the epithelial‐protective effects, pan depletion of IMφs using clodronate‐containing liposomes exacerbated epithelial injury in colitic mice.^59^, ^85^, ^86^ Similarly, ablation of CX3CR1^+^ IMφs significantly aggravated IEC damage in *Citrobacter rodentium*‐infected mice.^87^ Blocking monocyte infiltration, however, by disrupting the CCL2/CCR2 interaction yielded contradictory results: either aggravating^17^, ^88^ or mitigating^89^ colitis. This phenomenon indicates that IMφs (at least various IMφ subsets) contain the colitogenic properties as well.

Indeed, inimically many inflammatory cytokines produced by IMφs undermine the normal function of IECs, thus leading to the increased paracellular permeability. The best‐characterized epithelial cytotoxic cytokine is TNF‐α, which disrupts the epithelial barrier through multiple mechanisms.^90^ For example, TNF‐α triggers apoptosis of IECs in a caspase‐8‐dependent manner.^91^ TNF‐α also increases epithelial permeability through inducing the internalization of a tight junction protein, occludin. Furthermore, TNF‐α is found to be synergistic with IFN‐γ to impair the integrity of the epithelial barrier via increasing the expression and enzymatic activity of myosin light chain kinase. This results in the induction of tight junction dysfunction in IECs.^92^, ^93^ In a macrophage–IEC coculture system consisting of Caco‐2 IEC cells, TNF‐α produced by THP‐1 macrophages accounted for the impaired the expression of junctional protein ZO‐1 and E‐cadherin.^94^ In terms of the mucus barrier, TNF‐α administration induced goblet cell apoptosis in the intestine of infant mice, thereby contributing to the development of neonatal necrotizing enterocolitis.^95^ At present, the administration of several FDA‐approved anti‐TNF‐α monoclonal antibodies (e.g. etanercept, infliximab, adalimumab, certolizumab, golimumab) represents one of the most successful strategies in the clinical treatment of IBD.

Seemingly paradoxical, TNF‐α^–/–^ mice are more susceptible to DSS‐induced colitis. This genetic knockout exhibited higher numbers of inflammatory infiltrates as well as more severe mucosal damage compared to TNF‐α^+/+^ littermates.^96^ Similarly, TNF‐α^–/–^ mice showed impaired activation of Wnt/β‐catenin signaling in intestinal stem cells, which led to the reduced IEC proliferation and enhanced IEC apoptosis in colitic mice.^97^ Moreover, either TNFR1 or TNFR2 deficiency exacerbated colitis in mice.^98^ In fact, TNF‐α exerts certain epithelial‐protective functions. For instance, in IECs, TNF‐α was protective against apoptosis by transactivating the ErbB4 kinase, a process dependent on TACE‐mediated heparin‐binding EGF‐like growth factor (HB‐EGF) release.^99^ In addition, TNF‐α triggered COX2 expression in IECs in an EGFR‐dependent manner, initiating antiapoptotic signaling.^100^ TNFR2 signaling was also reported important for supporting IEC proliferation in colitic mice.^101^ Low levels of TNF‐α promoted ICE proliferation and accelerated wound closure of the IEC monolayer through a TNFR2 signaling‐dependent manner.^102^ Regarding the mucus barrier, TNF‐α promoted mucin secretion by either up‐regulating *MUC2* (validated through mRNA expression) by IECs,^103^ or inducing goblet cell differentiation.^104^ Additionally, TNF‐α also increased the number of chromogranin A‐expressing enteroendocrine cells.^105^

The multifaceted functions of TNF‐α are partially due to two factors: TNF‐α confers both prosurvival and proapoptotic signaling in IECs, which is highly dependent on its concentration, receptor selectivity, and downstream signaling elements.^106^ High concentrations of TNF‐α preferentially activated TNFR1 signaling, which resulted in a death receptor‐like state, thereby initiating caspase‐8‐dependent cell apoptosis. Concomitantly, TNFR1 engagement activated TRADD/TRAF2(or TRAF5)/NF‐κB pathway, which conferred a prosurvival signal. Conversely, low concentrations of TNF‐α preferentially bound to the alternative receptor, TNFR2, leading to the activation of either TRAF1 (or TRAF2)/NF‐κB pathway. This alternative signaling pathway enhanced cell proliferation and was found to mediate murine colitis. Complexifying matters further, soluble TNF‐α and membrane‐bound TNF‐α have independent, distinct bioactivity. Membrane‐bound TNF‐α can trigger a reverse signaling in macrophages to down‐regulate their production of inflammatory cytokines in a TGF‐β‐dependent manner.^107^ Hence, current knowledge about the specific roles of TNF‐α in intestinal inflammation may just be the tip of the iceberg. Although several anti‐TNF‐α therapies exist, approximately 1 out of 3 patients with IBD fail to respond to treatment,^108^ highlighting the dire need to identify other candidate colitogenic cytokines.^109^

It has been reported that high levels of IL‐18 were also associated with patients with IBD who had poor prognosis with anti‐TNF‐α therapies.^109^ Unsurprisingly, IL‐18 is heavily involved in colitis pathology. IMφ‐produced IL‐18 aggravated TNBS‐induced colitis.^110^ In agreement with these findings, researchers showed that blocking IL‐18 bioactivity using rhIL‐18BPa or anti‐IL‐18 reduced the severity of both TNBS colitis^111^ and DSS colitis,^36^ respectively. In contrast, the anticolitic effect of IL‐18 has also been reported.^112^ The impact of IL‐18 on the gut microbiome equilibrium^113^, ^114^ and/or the broad spectrum of cell types IL‐18 may act upon complicates the exact determination of IL‐18′s function in colitis. A more precise study using IEC‐specific IL‐18/IL‐18R1‐deficient mice and IL‐18 bp‐deficient mice revealed that IL‐18 derived from endothelial cells and/or hematopoietic cells (presumably including IMφs) aggravated DSS colitis development by disrupting goblet cell maturation.^115^ The goblet cell‐specific effect of IL‐18 indicates that each IEC population may possess distinct susceptibilities to IL‐18 signaling. In support of this, IMφs stimulated by gut bacteria produced prostaglandin, resulting in preferential disruption of normal goblet and Tuft cells, leading to an overall immunocompromised mucus barrier.^116^ Similarly, other macrophage‐produced IEC cytotoxic mediators include: IL‐6,^117^ reactive oxygen species (ROS),^118^ NO,^119^ and IL‐1β^120^ to mention a few. Many of the mediators are also considered “weapons” against pathogens.

It should be noted that even therapeutic approaches aiming to restore IEC barrier may also increase the risk of epithelial carcinogenesis. This can be elucidated by the fact that many factors facilitating IEC repair or proliferation also contribute to cancer tumorigenesis. For instance, overactivation of Wnt/β‐catenin signaling, a known anti‐inflammatory signaling cascade, is a key event in the initiation of colon carcinogenesis. Several mechanisms for the oncogenic roles of Wnt/β‐catenin signaling have been proposed, including: (1) sustaining the stemness of colon cancer stem cells,^121^ (2) promoting Th17 cell‐mediated inflammation,^122^ or (3) triggering chromosomal instability in the intestinal epithelium.^123^ A similar example is PGE2, a naturally occurring prostaglandin with oxytocic properties that accelerated mucosal healing while promoting the proliferation of colon cancer cells.^124^, ^125^ Despite possessing inflammation‐surpassing properties, most anti‐inflammatory effectors (e.g., IL‐10, TGF‐β, IL‐4, etc.) also dampen the macrophage‐mediated antibacterial immunity,^126^ which is a critical step for pathogen clearance. Therefore, the use of immunosuppressants for clinical‐based therapies should be heeded with caution, specifically in infection‐induced intestinal inflammation.

Together, redirecting inappropriate IEC‐IMφ crosstalk is of great significance for the rebuilding of barrier homeostasis and immune homeostasis in intestinal inflammation (Figure 1). Though specific murine genetic models have been created to model these questions, the commonly used conditional knockout mice, *Villin* ^Cre^ or *LysM* ^Cre^, are not exclusive enough for the precise examination of subset‐specific crosstalk between IECs and IMφs. To overcome this limitation, the development of more specific animal models is required.

## 4 CROSSTALK BETWEEN ENTERIC NERVOUS SYSTEM AND IMφS

### 4.1 Neuroregulation of IMφ function

**FIGURE 2:**
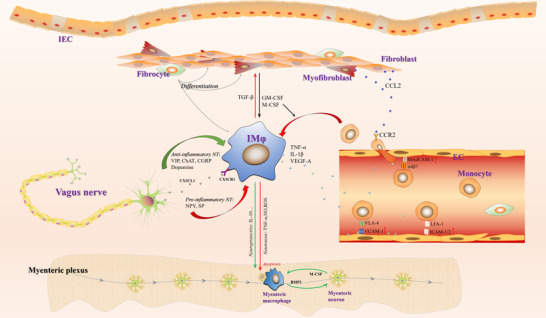
IMφ interplay with enteric neurons and mesenchymal cells

The mammalian intestinal tract is equipped with millions of neurons and nerve endings comprising the largest autonomic nervous system in the body. The intestine is therefore commonly regarded as our “second brain,” which works in partial independence of the CNS.^127^ Apart from its function in controlling motility and secretion of the intestinal tract, the enteric nervous system (ENS) is widely involved in modulating intestinal immunity.^128^ Enteric neurons and extrinsic nerve endings innervate the submucosal plexus, myenteric plexus, and lamina propria in the intestine. Curiously, these neurons are located in close proximity to IMφs, which express a broad range of receptors for neurotransmitters. Significant evidence has been reported to elucidate the neuron‐macrophage crosstalk and its physiologic relevance to the intestinal inflammation (Figure 2).

In the steady‐state intestine, the ENS is important in shaping the function of muscularis macrophages, which closely contact the myenteric plexus. Unlike the proinflammatory properties of lamina propria macrophages, muscularis macrophages mainly exert a tissue‐protective function. The ENS‐mediated macrophage reprogramming is further substantiated by the observation that either peritoneal macrophages or RAW264.7 macrophages cocultured with enteric neurospheres acquired some phenotypic features of muscularis macrophages. This change is similarly dependent upon the activation of adrenergic signaling in macrophages.^129^

The impact of the ENS on IMφs in intestinal inflammation is multifaceted. The most well‐known characterized model is the cholinergic anti‐inflammatory pathway (CAIP).^130^ Activation of the vagus nerve activity either by electrical stimulation or CNI‐1493 administration suppressed the inflammatory activity of macrophages.^131^ Compared with sham‐operated mice, vagotomized mice exhibited significantly higher levels of TNF‐α, IL‐1β, and IL‐18 in the inflamed colon. Importantly, macrophage function is indispensable for the anticolitic role from vagus nerve stimulation as vagotomy failed to exacerbate colitis in macrophage‐deficient mice.^132^ In a surgery‐induced intestinal inflammation model, VNS induced local secretion of acetylcholine, leading to decreased calcium transients and reduced proinflammatory activity in CX3CR1^+^ muscularis macrophages in the small intestine.^133^ An anatomical study demonstrated that the vagus nerve does not have a direct interaction with IMφs. Conversely, they exist in close contact with enteric neurons, which express vasoactive intestinal peptide (VIP) and choline acetyltransferase. These findings suggest that these specific mediators might be responsible for the anti‐inflammatory effects of VNS.^134^ Indeed, VIP dampened macrophage production of inflammatory cytokines by down‐regulating the NF‐κB pathway.^135^, ^136^ During the onset of intestinal inflammation, the expression of VIP in nerve fibers was significantly reduced.^137^ Supplementation of VIP alleviated TNBS colitis in mice, which was accompanied by a reduced number of IMφs as well.^138^, ^139^ The expression of TLR2/TLR4 on IMφs was also found to be down‐regulated by VIP.^140^ These findings may be helpful for maintaining macrophage hyperresponsiveness toward bacterial stimuli. Although these data suggest the protective role of VIP in intestinal inflammation, a contradicting report demonstrated that VIP exacerbated DSS‐induced colitis. In this particular study, mice receiving VIP antagonists exhibited lower levels of IL‐6, IL‐1β, and reduced disease activity.^141^

The effect of CAIP has been proposed to associate with vagus nerve‐mediated activation of sympathetic nerve fibers.^142^ IBD patients were found to have a reduced number of sympathetic neurons and their products, including noradrenaline, dopamine, and serotonin.^143^

In both DSS‐challenged mice and steady‐state IL‐10^–/–^ mice, sympathetic nerves were protective in a chronic colitis model.^144^ Consistently, noradrenaline and/or dopamine treatment suppressed TNF‐α production by macrophages in response to TLR ligand stimulation, thus restraining colitic progression.^145^, ^146^ It is worth mentioning an opposite result was reported, showing that chemical sympathectomy ameliorated colitis, while capsaicin‐induced activation of sympathetic nerves aggravated disease severity.^147^ This discrepancy may have arisen from the receptor‐specific effects of sympathetic neurotransmitters. Among other signaling pathways, activation of β‐adrenergic receptors induced an anti‐inflammatory signal in macrophages.^148^, ^149^ In contrast, the activation of α‐adrenergic receptor amplified inflammation.^149^, ^150^, ^151^ The cell type‐specific responsiveness to sympathetic neurotransmitters further complicates this problem. Recently, one study reported that sympathetic denervation, or sympathectomy, induced spontaneous colitis in *Rag1* ^−/−^ mice, evidenced by the increased number of inflammatory monocytes and elevated production of proinflammatory cytokines. Phenotypically, these studies suggest sympathetic innervation may be involved in suppressing innate inflammation.^152^

The proinflammatory neurotransmitter can be exemplified by NPY, a 36‐AA neuropeptide, which is expressed by myenteric neurons and submucosa neurons in the intestine. NPY deficiency decreased the production of TNF‐α and IL‐12 in macrophages challenged with various TLR ligands.^153^ Mice deficient in NPY, or its canonical receptor Y1, were less susceptible to either DSS‐induced colitis or *Salmonella* infection.^154^ The colonic release of neuropeptide Substance P (SP) by enteric neurons was increased in both TNBS and DSS‐challenged mice; SP deficiency protected mice from colitis, indicating a proinflammatory role of SP. Another neuropeptide, calcitonin gene‐related peptide, coreleased with SP during colitis, surprisingly exerted an anti‐inflammatory function.^155^ Similarly, SP release by lumbar dorsal root ganglia, was augmented in rat ileum after *Clostridium difficile* toxin A injection. Further supporting the protective phenotype, blocking SP function decreased TNF‐α production by toxin A‐stimulated IMφs.^156^

The communication with IMφs is also critical for the proper function of the ENS. Muscularis macrophages contribute to the development of the ENS by actively phagocytizing dying myenteric neurons.^157^ BMP2, produced by muscularis macrophages, was reported to modulate gastrointestinal motility by activating BMPR signaling in enteric neurons. The ablation of muscularis macrophages resulted in abnormal muscle contraction and slower intestinal transit time. In turn, enteric neurons secreted the growth factor M‐CSF to support further development of muscularis macrophages.^158^ In TNBS colitis, the number of muscularis macrophages was markedly increased with altered morphology. These macrophages were distributed around the interstitial cells of Cajal in the myenteric plexus and led to the intestinal dysmotility.^159^ *Salmonella Typhimurium* infection caused the death of intrinsic enteric neurons and reduced intestinal motility, whereas muscularis macrophages with the activated β2 adrenergic receptor signaling prevented infection‐induced neuronal loss. This protective effect was lost in macrophage‐depleted mice but remained intact in CCR2^–/–^ mice. This phenotype is highly suggestive that macrophages play a negligible role in neuroprotection.^160^ In contrast, many byproducts from inflammatory macrophages are neurotoxic: TNF‐α, NO, and ROS, for example.^161^, ^162^, ^163^ Recent studies identified two distinct “microglia‐like” IMφ subsets—one that resides around the enteric ganglia with a CD45^+^ChB6^+^MHC‐II^+^ phenotype, whereas the other is located primarily within the intestinal submucosa and muscularis externa. While the function of these intraganglionic CD45^+^ChB6^+^MHC‐II^+^ macrophages is unknown,^164^ the other subset possesses a unique self‐renewal capacity. Furthermore, these embryo‐derived IMφs retained a similar gene signature to microglia and were responsible for the maintenance of the number and secretory function of enteric neurons,^11^ which mimicked the supportive function of microglia on central neurons.

### 4.2 Separated brothers? IMφs and microglia

Intriguingly, among all reported tissue‐resident macrophages, IMφs might inherently possess a more analogous gene expression profile to microglia,^165^ thus engendering the term “microglia‐like” macrophages.^166^ Various microglia‐specific genes are also highly expressed in IMφs, including *Cx3cr1*, *Mertk*, *Gas6*, *Fcrls*, and *P2ry12*, yet these unique gene signatures are not shared by the macrophage populations located within the lung, skin, peritoneum, or spleen. Furthermore, transcription factors such as *Atf3*, *Junb*, and *Egr1* exhibit high expression only in microglia and IMφs, but not in other tissue‐resident macrophages This expression profile is indicative of the similar transcriptional basis underlying their identity.^165^, ^166^ The sizeable similarity is quite interesting because the microenvironment differs between the brain and the gut. The gut mucosa is an “open” interface, exposed to a vast quantity of microbial and food antigens, therefore possessing a dense vascular system to deliver circulating leukocytes into the gut. In contrast, the brain is a relatively “isolated” tissue due to the existence of the blood–brain barrier, which prohibits the entrance of most leukocytes. More than likely, the shared gene profiles between IMφs and microglia might have arisen to reflect their common need in scavenging apoptotic cells, repairing damaged tissues, and clearing invading pathogens all while inducing minimal inflammatory responses. In fact, existing equivalent regulatory mechanisms between microglia and IMφs have been elucidated. For example, both IMφs and microglia express high levels of CX3CR1, whereas expression is remarkably lower—and in some cases—undetectable in tissue‐resident macrophages. In neuronic inflammation, CX3CR1 ligation by the CNS‐derived CX3CL1 decreased inflammatory cytokine release from LPS‐activated microglia. In turn, the reduced production of TNF‐α by microglia alleviated their neurotoxic profile.^167^ Likewise, neutralization of CX3CL1 augmented the levels of TNF‐α and 8‐isoprostane in rat hippocampi.^168^ CX3CR1 deficiency further exacerbated neuronal loss in both Parkinson's and amyotrophic lateral sclerosis murine models.^169^ These reports demonstrate that the neuronal‐CX3CL1/microglial‐CX3CR1 axis exerts crucial anti‐inflammatory functions in the CNS. In the intestine, mice deficient in either CX3CR1 or CX3CL1 exhibited severe colitis in comparison to littermate controls, due to the decreased number of IMφs and enhanced commensal bacteria translocation.^65^, ^170^ Further mechanistic insights elucidated that these CXCR3^–/–^ mice displayed markedly blunted production of IL‐10 by IMφs as well, resulting in impaired proliferation of Tregs and consequentially disrupting oral tolerance.^170^

Collectively, neurotransmitters do possess similarities with traditional cytokines in regulating IMφ functions, yet in independent mechanisms. Their actions are generally swifter, henceforth many neurotransmitters have already been synthesized and stored in resting neurons. Because of their short half‐life, neurotransmitters often cover a relatively short action distance, mainly affecting neighboring cells. Additionally, the production of neurotransmitters is afflicted by stress, anxiety, fear, pain, and/or depression. Future efforts should be more vigilant in deciphering how mental discomfort modulates the function of IMφs, and in return, the manner of IMφ feedback to neuronal signals.

## 5 CROSSTALK BETWEEN MESENCHYMAL CELLS AND IMφS

Intestinal mesenchymal cells are comprised of multiple cell types besides epithelial cells and immune cells in the intestine. These heterogeneous cell populations mainly include fibroblasts, myofibroblasts, fibrocytes, and endothelial cells, which together maintain the intestinal structure and constitute the vascular system within the intestinal stroma (Figure 2).

Mesenchymal cells can sense various environmental cues upon which they generate immunoregulatory signals to alter IMφ functions. For example, supernatant from intestinal stromal cell culture, but not that from IECs or lamina propria cells, induced the differentiation of peripheral monocytes into mature IMφs. In terms of function, stromal cell culture supernatant decreased the production of inflammatory cytokines through activation of monocytes and macrophages in a TGF‐β‐dependent manner.^15^ In *C. rodentium‐*infected *mice*, colonic stromal cells produced high levels of CCL2 to attract Ly6C^hi^ monocytes, promoting the eradication of *C. rodentium* in the colon. In contrast, IECs and colonic CD11b^+^ myeloid cells produced less CCL2 in the same context.^171^ In this work, the effector stromal cells were regarded primarily as fibroblasts.

### 5.1 IMφ–fibroblast interplay

Fibroblasts from patients with IBD had both enhanced proliferation and activation compared with those from healthy donors.^172^ Upon activation, these fibroblasts secreted a set of macrophage growth factors such as M‐CSF and GM‐CSF. This secretory function might affect the differentiation, polarization, and survival of IMφs.^173^, ^174^, ^175^ In turn, MyD88 signaling in IMφs mediated the enrichment of COX‐2^+^ stromal cells, most of which are fibroblasts located around colonic crypts of DSS‐treated mice. This macrophage‐fibroblast interplay promoted epithelial repair after mucosal damage in a PGE2‐dependent manner.^176^ Moreover, IMφ‐derived IL‐36α protected mice from DSS colitis partially by activating IL‐36R signaling in fibroblasts, therefore promoting mucosal healing.^77^

As an activated form of fibroblasts, colonic myofibroblasts are potent producers of various macrophage‐modulatory cytokines in response to inflammatory stimuli, such as: CCL2, IL‐6, M‐CSF, and TNF‐α.^177^, ^178^ Recent evidence demonstrated that myofibroblasts‐derived osteopontin increased M2 polarization of IMφs via binding to α_v_β_3_ and CD44.^179^ Reciprocally, IL‐13‐stimulated macrophages produced TGF‐β to promote myofibroblast activation.^180^ Enhanced production of Wnt ligands from CD16^+^ macrophages in STAT6^–/–^ mice led to the abnormal accumulation of fibroblasts and myofibroblasts, resulting in aggravation of intestinal fibrosis in a TNBS chronic colitis model.^181^ These results suggest that despite their contribution to epithelial healing, the excessive activation or accumulation of fibroblasts/myofibroblasts can potentially lead to intestinal fibrosis, a nearly irreversible disease that may cause permanent intestinal dysfunction in IBD patients. Macrophage functions are closely intertwined with intestinal fibrosis prognosis^182^, ^183^; however, this topic is beyond the scope of the current article. Still, it is interesting to note that myofibroblasts were reported to be transdifferentiated from CD68^+^ macrophages in renal fibrosis. Whether this phenomenon also occurs in colitis, the microenvironmental impact and its physiologic significance remain to be further explored.^184^, ^185^, ^186^

Another subpopulation under the blanket of hematopoietic‐derived cells are fibrocytes. These unique cells are circulating precursors for fibroblasts/myofibroblasts and have also been shown to exist upstream of certain kinds of immune cells. These bone marrow‐derived cells coexpress markers for stem cells (CD34), leukocytes (CD45), and myofibroblasts (α‐SMA), and can also migrate to inflammatory sites, upon which they have the propensity to differentiate into fibroblasts, macrophages, endothelial, or epithelial cells depending on the environmental cues.^186^ In the presence of M‐CSF, fibrocytes differentiated into CD11b^+^F4/80^+^ macrophages with high phagocytic capacity; meanwhile, in the presence of GM‐CSF, they preferentially differentiated into CD11b^+^CD11c^+^ DCs.^187^ Serum deprivation‐induced monocyte‐to‐fibrocyte transition was a process amplified by IL‐4 and IL‐13 yet was inhibited by IFN‐γ and serum amyloid P.^188^, ^189^, ^190^ Fibrocytes themselves serve as important sources of immunoregulatory cytokines including; CCL2, TNF‐α, IL‐10, TGF‐β, and so on.^191^ In addition to macrophages, fibrocytes can also transdifferentiate into fibroblasts or myoblasts, by which they participate in wound healing.^185^, ^192^ The pluripotency characteristic of fibrocytes compels several interesting questions. First, what is the overall impact of fibrocytes on different kinds of intestinal inflammation? Second, are there phenotypic and functional differences between fibrocyte‐derived macrophages and monocyte‐derived macrophages in the intestine? Third, how do various cytokines or environmental factors affect fibrocyte differentiation in the inflamed intestine? Moreover, can the fibrocyte differentiation process be manipulated to treat intestinal inflammation? Taken together, the knowledge on the roles of fibroblast lineage in intestinal inflammation are quite limited to date. Considering the widely regulatory functions of these cells in immune regulation,^193^ their interaction with IMφs is undoubtedly worth further exploration.

### 5.2 IMφ–endothelium interplay

Recently, the importance of endothelial function in colitis pathogenesis has been gaining attention. The vascular and lymphatic endothelium link the inflamed colon with blood and lymphoid organs by which they control the entry or exit of leukocytes, bacteria, and chemokines. Patients with severe IBD typically are found to have significant endothelial dysfunction. For instance, intestinal vascular endothelial cells from patients with IBD exhibited increased expression of VCAM‐1 and ICAM‐1/2,^194^ both of which are crucial for the adhesion of circulating leukocytes (including monocytes). The elevated levels of these adhesion molecules are partially a byproduct from the excessive TNF‐α production by IMφs. Treatment of patients with IBD with an anti‐TNF‐α monoclonal antibody normalized VCAM‐1 and ICAM‐1/2 expression on intestinal endothelium.^195^, ^196^

Interference of endothelium adhesion of leukocytes has proven very effective in clinical IBD treatment. Vedolizumab, an FDA‐approved drug, prevents the infiltration of α4β7‐expressing T cells into the inflamed colon by blocking α4β7 binding to its endothelial ligand MAdCAM‐1. Surprisingly, in a very recent study, vedolizumab administration was found to have negligible effect on the number of intestinal CD4^+^ T cells, CD8^+^ T cells, and memory T cells; nor did vedolizumab obviously alter levels of the T cell activation markers CD69/CD25. Instead, vedolizumab dramatically reduced the number of M1 macrophages while simultaneously increasing the number of M2 macrophages in patients with IBD.^197^ This finding was quite unexpected. Although α4β7 integrin is involved in monocyte adhesion, its blockage was traditionally thought to preferentially disrupt T cell recruitment.^198^ More than likely, there exists a compensatory mechanism involving T cell trafficking into the inflamed intestine. This work highlights the potential therapeutic significance of interfering with monocyte–endothelium interactions. In turn, the enhanced MAdCAM‐1 expression may be attributed to IMφ dysfunction. TNF‐α and IL‐1β were reported to induce MAdCAM‐1 expression on both human and mouse endothelial cells.^199^ Also, both NF‐κB and PI3K/Akt signaling were necessary for this process in intestinal vascular endothelial cells.^200^

The progression of intestinal inflammation is often accompanied by pathologic angiogenesis, which in turn perpetuates inflammation to form a seemingly vicious repeating cycle.^201^ Compared with healthy individuals, patients with poor IBD prognosis often exhibited higher densities of blood vessels in their intestines.^202^ Macrophages play pivotal roles in modulating abnormal angiogenesis processes in intestinal inflammation. Upon sensing angiogenic signals (such as hypoxia), macrophages migrated to the site of neovessels, secreting proangiogenic cytokines, including NO or varying proteases to either stimulate endothelial cell proliferation or provide a favorable niche for neovessel growth.^201^ In colitis, macrophage‐derived VEGF‐A increased disease susceptibility by disrupting endothelium function.^203^ On the other hand, IMφ–endothelium interactions were also reported to be protective in colitis. For example, IMφs were crucial for maintaining the gut homeostasis by preventing the leakage of the vascular endothelium.^11^ Moreover, macrophage‐derived HB‐EGF preserved villous blood flow and microvascular architecture, thereby ameliorating necrotizing enterocolitis. In addition to acting on endothelial cells, dermal macrophages can differentiate into pericytes, which were found to be pivotal in maintaining the survival and function of endothelial cells.^204^ Whether this trans‐differentiation process also occurs in the context of intestinal inflammation remains to be validated.

In conclusion, although mesenchymal cells are traditionally thought of as being irrelevant compared to IECs in affecting IMφ functions (Figure 2), emerging clinical evidence has suggested that targeting the mesenchymal cell–IMφ interaction in fact provides benefits in alleviating intestinal inflammation.

## 6 MULTIPLE PLAYERS—HIGHLY INTERTWINED CROSSTALK

Although many delicate models depicting intracellular communication have been proposed, the actual physiologic microenvironment in the intestine is far more complex. In many circumstances, IMφs interact with nonhematopoietic cells through a “third party,” which can be either adaptive immune cells, innate lymphoid cells, or gut microbiota.^205^ One of the fundamental roles of macrophages is to modulate adaptive immunity, corresponding to a profound impact on the pathologic processes of intestinal inflammation. For instance, macrophages are important sources of several well‐known Th17 cell‐inducing cytokines (IL‐6, TGF‐β, IL‐1β) or Th17 cell‐maintaining cytokines (IL‐23). In a similar manner, macrophages regulate the differentiation of type 3 innate lymphoid cells (ILC3). IL‐17A produced by Th17 cells and ILC3s are crucial for maintaining epithelial integrity through preventing the internalization of the tight junction protein occludin in IECs.^206^, ^207^ In *C. rodentium*‐induced colitis, deletion of CX3CR1^+^ macrophages resulted in reduced secretion of IL‐22 by innate lymphoid ILC3, leading to the decreased production of AMPs in colonic epithelium and delaying colonic clearance of *C. rodentium*.^208^ In addition to affecting T cell or ILC polarization, macrophages are the main sources of various T cell chemokines, including: Th1 cell chemoattractants CXCL9/CXCL10/CXCL11,^209^, ^210^, ^211^, ^212^ Th2 cell chemoattractant CCL24,^213^ Th17 chemoattractant CCL20,^214^ and Treg cell chemoattractants CCL17/CCL22.^215^, ^216^ In this manner, macrophages selectively recruit different T cell subsets, which then orchestrate the many functions of IECs and stromal cells in independent manners.

The communication between IECs and IMφs is often bridged by gut microbiota. IECs and their products play crucial roles in controlling the number, species, and distribution of the gut microbiota^27^ Increased permeability of the epithelial barrier permits the invasion of gut bacteria into the lamina propria, resulting in inflammatory activation of IMφs.^217^ Nevertheless, appropriate signals from the gut bacteria are also required for the functional equilibrium of IMφs. Compared with IMφs isolated from specific pathogen‐free mice, IMφs from germ‐free mice had impaired IL‐10 production in the resting state yet produced markedly higher levels of TNF‐α and IL‐6.^218^ It is uncertain whether the microbiota themselves or their metabolites prime the function of IMφs. Perchance, IECs are involved in microbiota‐induced macrophage priming by providing a selectively permeable barrier, permitting transportation of the appropriate microbial information to IMφs at the basolateral side. This process must be subjected to delicate regulation in order to maintain homeostatic microbiota. In a more complex model, gut microbiota‐stimulated IMφs secreted IL‐1β, which in turn drove the production of GM‐CSF in ILC3s. ILC3‐derived GM‐CSF was then found to induce the generation of regulatory IMφs and DCs, prompting promotion of Treg differentiation. Tregs, together with regulatory IMφs and DCs, produced IL‐10, which was involved in maintaining IEC barrier and immune tolerance.^219^ In fact, the intracellular communication in the intestine is often mediated by soluble cytokines/peptides in a paracrine manner, alluding that multidirectional crosstalk is conceivably the most common way that cell functions are modulated. The interplay among the immune system, epithelial system, microbial system, and nervous system are summarized in many previous reviews^220^, ^221^, ^222^, ^223^; therefore, we will not discuss these topics in further detail.

## 7 THERAPEUTIC IMPLICATIONS—FROM A MACROPHAGE PERSPECTIVE

Although in cancer treatment, global depletion of tumor‐associated macrophages has proven to be a feasible strategy.^224^ Ablation of IMφs indistinguishably aggravates intestinal inflammation due to their indispensable roles in mucosal repair, bacterial clearance, and tissue remodeling. In this regard, in‐depth dissection of IMφ subpopulations and their unique functions is necessary for precise therapeutic intervention.

As described above, the administration of CCL25‐conjugated sepharose or vedolizumab can prevent the entry of peripheral monocytes into the lamina propria; however, these cells are also strong fighters against the invading pathogens. For example, inflammatory monocytes mediated the clearance of *C. rodentium* in a colitis model. Reduced CCL2 production impaired the colonic infiltration of inflammatory monocytes, leading to the enhanced bacterial burden in mice.^171^ Therefore, the dichotomy of whether we should reject CCL2 to reduce inflammation at the expense of their bactericidal activity needs careful consideration. This becomes particularly important in patients with infection‐induced intestinal inflammation.

The continuous replenishment of IMφs from peripheral blood results in their inefficient ablation in patients with IBD. This sheds light on another important biologic process—recruitment and accumulation of myeloid‐derived suppressor cells (MDSCs) in tumor tissues. Similar to intestinal inflammatory monocytes, MDSCs are also immature myeloid progenitors but with immunosuppressive properties. The differentiation of MDSCs into mature macrophages is impaired in the tumor microenvironment, similar to the disruption of maturation of Ly6C^hi^ inflammatory monocytes in the inflamed gut.^225^, ^226^ Owing to the high phagocytic and low inflammatory properties of mature CX3CR1^hi^ macrophages, guiding the differentiation of mature IMφs from their inflammatory progenitors may redirect them into an anticolitic phenotype. It is reported that TNF‐α disrupts the differentiation of monocytes into macrophages during *Mycobacterium tuberculosis* infection.^227^ Accordingly, TNF‐α neutralization in IBD patients decreased the number of CD14^hi^ monocytes while simultaneously increasing the number of CD206^+^ M2‐like macrophages.^228^ Another proinflammatory cytokine IFN‐γ exerts a similar inhibitory effect on macrophage differentiation in colitis.^229^ Some neurotransmitters are involved in the differentiation process, too. VIP inhibited the transcription factor PU.1 and the level of the M‐CSF receptor on monocytes.^230^ In colitic mice, sympathetic denervation increased the ratio of inflammatory monocytes to resident macrophages.^152^ Finally, endothelium and vascular dysfunction may also be involved in macrophage differentiation by affecting oxygen accessibility. It was reported that a hypoxic microenvironment promoted macrophage differentiation from MDSCs.^231^

Apart from the differentiation status, current evidence suggests that the localization of IMφs is closely associated with their phenotypes and functions, with subepithelial IMφs considered generally proinflammatory, while those located in the deeper layers of the intestine mainly possessing tissue‐repairing properties. It is uncertain whether the fate of IMφs is already predetermined before they enter the gut, or if it is dictated by certain intratissue chemoattractive signals. More than likely, the distribution and permeability of blood vessels and the expression of adhesion molecules on endothelial cells is necessary in controlling the site of monocyte influx.

Blocking inflammatory cytokines is one of the most popular strategies in clinical IBD treatment. A typical example is the class of anti‐TNF‐α antibodies. Other promising candidates include antibodies against IL‐17A, IL‐23, IL‐18, and other various proinflammatory cytokines.^115^, ^232^, ^233^, ^234^ It is worth mentioning that this approach leads to mild to severe side effects in patients with IBD. The blockage of these cytokines has the propensity to compromise their protective effects as well. It is better to evaluate the functional status of the intestine for each patient with IBD to achieve personalized treatment. For example, blocking IL‐18 may be particularly beneficial in patients with IBD with massive goblet cell loss. Administration of immunosuppressive cytokines are also a viable therapeutic option. IL‐10, due to its anti‐inflammatory abilities and promotion of IEC repair, has been proposed to have therapeutic potential. IL‐10 therapy was shown to be protective against colitis progression in many animal studies with no obvious side effects reported.^235^, ^236^, ^237^, ^238^ However, administration of recombinant human IL‐10 (Tenovil) in patients with IBD yielded inconsistent therapeutic effects among different clinical trials. Patients exhibited improved colitis symptoms in three trials,^239^, ^240^, ^241^ juxtaposed to two other trials where IL‐10 supplementation failed in alleviating colitis.^242^, ^243^ Further optimization of IL‐10‐based therapy is hindered by the lack of knowledge on how IL‐10 signaling is regulated in the intestine.^244^ A recent study reported that TNF‐α increased macrophage expression of phosphatase Shp2, which exacerbated colitis by desensitizing macrophages to the anti‐inflammatory function of IL‐10. This finding suggests that TNF‐α neutralization may act synergistically with IL‐10 administration to exert a “double strike” on macrophage‐mediated intestinal inflammation.

In addition to cytokine‐based treatments, various bacterial metabolites are also utilized to correct the inappropriate functions of IECs and IMφs in intestinal inflammation. Butyrate, a product of microbial fermentation, mainly metabolized in IECs, is beneficial for the maintenance of the epithelial barrier by increasing the expression of mucin 2,^245^ AMP LL‐37,^246^ and several tight junction proteins.^247^ This short‐chain fatty acid also inhibited the inflammatory activation and promoted M2 polarization of macrophages.^248^, ^249^, ^250^

In terms of a signaling pathway‐based approach, distinct cell‐specific responsiveness may make the therapeutic outcome unpredictable. For example, many pathogenic cytokines proceed through JAK/STAT signaling such as IL‐13, IL‐23, and IFN‐γ; therefore, JAK inhibitors (e.g., Tofacitinib) are clinically used in IBD treatment.^251^ A latest work reported that Tofacitinib corrected the pathogenic IEC–IMφ interaction induced by loss of *PTPN2*.^252^ Unfortunately, JAK inhibition also blocks some anti‐inflammatory or barrier‐protective pathways, such as IL‐10/STAT3, IL‐22/STAT3, and IL‐4/STAT4 pathways. For example, an intriguing dichotomy exists with the STAT3 signaling pathway: its activation in IECs^253^, ^254^ or IMφs^255^ is thought to be anticolitic, whereas its activation in T cells exacerbates colitis.^254^, ^255^, ^256^ Another example is NF‐κB signaling, which is the predominant proinflammatory pathway in IMφs.^257^ Though implicated as a potential therapeutic target, it also plays a crucial role in the survival and proliferation of the injured IECs, complicating development of clinically relevant therapies.^258^

Therapeutic interventions via mesenchymal cell‐macrophage crosstalk disruption have also been reported. CD45^−^CD73^+^CD90^+^CD105^+^ intestinal mesenchymal cells blunted macrophage production of inflammatory cytokines in colitis.^259^ Furthermore, bone marrow mesenchymal stem cells (MSC) reduced severity of colitis through secretion of TSG6, facilitating the accumulation of IL‐10‐producing macrophages.^260^ In another work, the anticolitic role of MSCs was attributed to extracellular vesicles.^261^ Similarly, exosomes from umbilical cord mesenchymal stem cells (UCMSCs) were able to suppress the infiltration of inflammatory macrophages and reduce their production of colitogenic cytokines, thus alleviating DSS colitis in mice^262^ The exact component(s) responsible for the anticolitic effect of UCMSC‐derived exosomes still need to be further elucidated.

**FIGURE 3:**
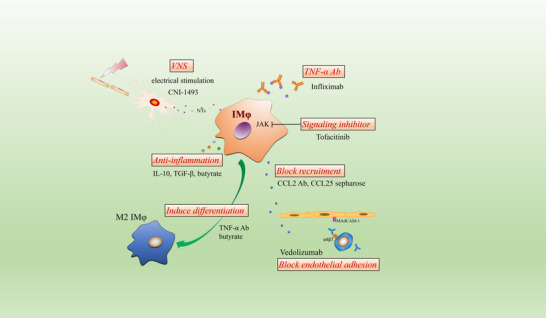
IMφ‐based therapeutic strategies for intestinal inflammation

In summary, for each individual patient, the type, dosage, frequency, and delivery route of therapeutics should be carefully considered and personalized to the patient in order to achieve a satisfactory therapeutic outcome with minimal degree of adverse side effects (Figure 3).

## 8 CONCLUDING REMARKS

Over the last few decades, significant progress has been achieved in understanding the phenotypes and functions of IMφs. Although researchers have a greater understanding now than ever before, perhaps we also must admit that the more we study IMφs, the more complex the cell type becomes. Here we can cite a resentence from Churchill, “There are no permanent enemies and no permanent friends, only permanent balance.” The traditionally regarded “bad guys,” such as colitogenic inflammatory cytokines and their producing cells, also serve their own unique function to maintain the intestinal equilibrium. Just like an advanced ecosystem, killing all “pests” will result in disrupted homeostasis. In this sense, further studies should be done to put more emphasis on how we can rebuild a balanced intestinal microenvironment. Although the heterogeneity and plasticity of IMφs pose many obstacles for investigators, this fortunately means IMφs are not so “stubborn”; there exist several undiscovered phenomena. To ultimately make IMφs more controllable, a deeper understanding into the mechanisms regulating intracellular communication is imperative.

## AUTHORSHIP

R. T. M., X. C., Q. C., and P. X. wrote the manuscript. R. T. M., K. N. S., and X. C. drew the figures. Q. C. and R. T. M. contributed equally to this work and share first authorship.

## DISCLOSURE

The authors declare no competing interests.
